# Editorial: Immune response to gram-negative bacteria in the lungs

**DOI:** 10.3389/fcimb.2024.1503892

**Published:** 2024-10-24

**Authors:** Agnes Jara-Collao, M Cecilia Poli, William Bain, Hernán F. Peñaloza

**Affiliations:** ^1^ Millennium Institute on Immunology and Immunotherapy, Facultad de Ciencias Biológicas, Pontificia Universidad Católica de Chile, Santiago, Chile; ^2^ Facultad de Medicina Clínica Alemana de Santiago, Universidad del Desarrollo, Santiago, Chile; ^3^ Programa de Inmunogenética e Inmunología Traslacional, Instituto de Ciencias e Innovación en Medicina, Facultad de Medicina, Santiago, Chile; ^4^ Unidad de Inmunología y Reumatología, Hospital Roberto del Río, Santiago, Chile; ^5^ Division of Pulmonary, Allergy, Critical Care, and Sleep Medicine, Department of Medicine, University of Pittsburgh, Pittsburgh, PA, United States; ^6^ Departamento de Laboratorios Clínicos, Escuela de Medicina, Pontificia Universidad Católica de Chile, Santiago, Chile

**Keywords:** gram-negative bacteria, acute pulmonary infection, chronic pulmonary infection, antimicrobial resistance, host immune response

Infectious diseases are a major cause of mortality and morbidity. The most common microbial pathogens accounted for nearly 1 in 7 of all deaths worldwide in 2019 (Collaborators, 2022). Although many infections can cause disease, a small handful of bacteria species account for nearly one-third of all infection-related deaths. In 2019, the 5 most common bacterial species accounted for 7% of global death (Collaborators, 2022), three of which were Gram-negative bacteria. Although infection by Gram-negative bacteria can be fatal at many sites, infections of the lower respiratory tract are a particularly challenging global health burden. In response to this global health challenge, we provide a Research Topic that provide new and key research findings in the study of Gram-negative bacterial infections of the lung and the immune response elicited.

Pneumonia, a severe form of lower respiratory tract infection, is characterized by a disruption of gas exchange at the alveoli. Pneumonia is often classified as community acquired pneumonia (CAP) or hospital acquired pneumonia (HAP) according to where it is contracted (Torres et al., 2021). Differences between CAP and HAP are not restricted to where the disease is acquired, but also in terms of etiology and patient risk factors. CAP is commonly caused by respiratory viruses (e.g. rhinovirus, respiratory syncytial virus, influenza, and coronoviridae) and bacteria (*Streptococcus pneumoniae*) (Jain et al., 2015), while HAP are caused mostly by Gram-negative bacteria such as *Klebsiella pneumoniae, Pseudomonas aeruginosa, Acinetobacter baumannii* and others (Jones, 2010). Factors that have been associated with increased risk of HAP are primarily associated with medical procedures (e.g. surgeries), hospitalization for acute diseases (acute respiratory distress syndrome), or underlying co-morbidities such as pre-existing lung disease (Torres et al., 2021). Better understanding of the risk factors that render susceptibility to HAP caused by Gram-negative bacteria is extremely important and highlights opportunities to prevent and manage these infections. One such study included in this Research Topic was conducted in 305 patients admitted to the intensive care unit at a large academic medical center in China. The authors evaluated risk factors associated with mortality during Gram-negative pneumonia, finding that lactic acid levels in circulation, tracheal intubation, and acute kidney injury were all independently associated with increased 30-day mortality during Gram-negative pneumonia (Liao et al.). Further studies are needed to validate these and other risk factors across different cohorts, as well as to determine whether any HAP risk factor may be treatable to improve patient outcomes.

In addition to the current global health burden of Gram-negative bacteria infections, there is a developing health crisis with the rise of elevated antimicrobial resistance (Torres et al., 2021) due to the lack of effective therapies and the high mortality associated with these infections. Epidemiological and genetic studies related to the acquisition of antimicrobial resistance by pathogenic bacteria are a highly dynamic field with importance to patient outcomes. For example, studying the acquisition of genes that confer resistance to last resort antibiotics such as colistin and carbapenems may help to prevent patient mortality, particularly in immune-suppressed patients (Zhang et al., 2022). In this Research Topic, Zhao et al. characterized an isolate of the Gram-negative bacterium *Klebsiella pneumoniae* (ST656) obtained from the broncho-alveolar lavage fluid of a lung transplant patient that co-harbored a plasmid-encoded betalactamase *bla*_NDM-5_ and *mcr-8.2* genes. While the *bla*_NDM-5_ gene was found to be located on an IncX3 type plasmid, the *mcr-8.2* gene was found within a conjugative plasmid pKP32558-2-mcr8. This isolate showed resistance to carbapenem, colistin, and tigecycline thereby rendering antimicrobial therapy to futility. The rise of a pan-resistant isolate from the acquisition of multiple antimicrobial resistance genes points to the evolution of “super bugs” that are of rising concern worldwide, which deserves close surveillance and the development of additional therapeutic strategies.

To develop novel therapies, it may be beneficial to study the host immune response to bacteria. For example, when a pathogenic bacterium reaches the alveolar space, epithelial cells and resident alveolar macrophages rapidly detect the presence of the pathogen through pattern recognition receptors (Nieto et al., 2013; Gonzalez-Ferrer et al., 2021). In conjunction, they initiate a coordinated response that involves the production of antimicrobial peptides, pro-inflammatory cytokines and chemokines required for the recruitment of neutrophils, monocytes and other immune cells relevant for host defense (Nieto et al., 2013; Penaloza et al., 2019; Gonzalez-Ferrer et al., 2021; Penaloza et al., 2021; van der Geest et al., 2023). The close interaction between alveolar macrophages and epithelial cells coordinates the subsequent responses in the lungs against bacteria. This interaction is summarized in a mini-review article in this Research Topic (Xue et al.) that describes the existence of multiple mechanisms by which host epithelial cells and alveolar macrophages interact with each other to modulate their pro- and anti-inflammatory profile required for host defense during infection with the bacterium *Mycoplasma pneumoniae*. These mechanisms of interaction include cytokine production (IL-1β, IL-23, TNF-α, IL-12, GM-CSF, IL-10, TGF-β), microRNAs and proteins expression such as the suppressor of cytokine signaling protein secreted through exosomes, surfactant-associated protein A (SP-A) and communication thought gap junction connexins. In addition, bacterial recognition by alveolar macrophages and lung epithelial cells initiates a response that facilitates the antigen uptake and presentation by dendritic cells to T lymphocytes (Nieto et al., 2013). The adaptive immune response mediated by effector T lymphocytes may differ by type of pathogen, which is summarized in another mini review by Gao et al. included in this Research Topic. For example, while a Th1 response is optimized against intracellular pathogens such as viruses, the Th2 response is efficient against parasites and the Th17 response is designed to respond against extracellular bacteria such as *Klebsiella pneumoniae*, *Pseudomonas aeruginosa*, *Acinetobacter baumannii*. Pathogenic bacteria have adapted to different components of the host immune system, which often result either in the disruption and evasion of the host immune response, or in cell death, tissue inflammation and injury (Bain et al., 2024). These adaptations may lead to chronic or recurrent infections (Gonzalez-Ferrer et al., 2021; Bain et al., 2024). Chronic infections caused by *P. aeruginosa* may induce an aberrant activation of T cells, leading to a Th2 response, characterized by being unable to clear *P. aeruginosa* and to amplify lung injury, promoting bacterial colonization instead of its clearance (Gao et al.). Importantly, chronic infections caused by *P. aeruginosa* and other Gram-negative bacteria are common in patients with lung structural diseases such as cystic fibrosis (CF). CF is a progressive disease caused by mutations in the gene that encodes the extracellular receptor cystic fibrosis transmembrane conductance regulator (CFTR) that modulates the transport of chloride to the airway lumen (Saint-Criq and Gray, 2017). Along with an altered mucus viscosity, CF patients normally suffer from recurrent infections by bacteria such as *Burkholderia cepacia*, which is an uncommon but frequently morbid cause of disease (Blanchard and Waters, 2019). As the immune response may vary from one patient to another, the identification of biomarkers is a useful tool to predict the efficacy of an immune response or disease severity. In this Research Topic, an original research article studied different biomarkers associated with disease progression in 116 pediatric CF patients, including 47 of whom were chronically infected with *Burkholderia cepacia* (Shmarina et al.). This study showed that *Burkholderia cepacia* infection was associated with reduced survival in CF patients. Interestingly, patients with *B. cepacia* were less likely to grow *S. aureus*, *P. aeruginosa*, or *Acinetobacter* species on sputum culture. Furthermore, they found elevated levels of the pro-inflammatory cytokine TNF-α in sputum samples. In contrast, they found reduced levels of IL-17F in both sputum and blood as well as lower levels of IL-18, TGF-β1 and IL-10 in blood. The mechanistic implications of these biomarkers in CF remain to be elucidated and constitutes an exciting area for future research.

As suggested by differences in co-infecting bacteria in children with CF with or without *B. cepacia* colonization, the complex interactions between the bacteria that colonize and/or chronically infect the lung airways and airspaces may have significant impacts on health and disease. Animal models have been useful to provide cellular and molecular insights with translational potential regarding the interaction between pathogenic bacteria, the microbiota and the host immune system during chronic infections. In an original research article in this Research Topic, Stoner et al. showed that in rats, the commensal microorganism *Streptococcus salivarius* may alter the glucose metabolism of *P. aeruginosa*, thus preventing *P. aeruginosa* colonization of the lung. Co-infection of *P. aeruginosa* in the presence of *Streptococcus salivarius* was associated with reduced expression of the pro-inflammatory cytokines IL-1β, IL-6, CXCL-2 and TNF-α as well as reduced lung immunopathology compared to rats infected with *P. aeruginosa* alone. *In vitro*, the co-culture of *S. salivarius* and *P. aeruginosa* in synthetic CF sputum media led to downregulation of *P. aeruginosa* genes involved in metabolism and reduced intracellular glucose highlighting an interesting pathogen-pathogen interaction that may have consequences for host-pathogen interactions and host biology.

Taken together, this Research Topic highlights the importance of the study of Gram-negative bacteria, the host immune response, and microbiota in the lung. Further work is needed to understand the microbiological and epidemiological fundamentals of Gram-negative bacteria infections, including antimicrobial resistance, and the risk factors associated with respiratory infections caused by these microbes. Better understanding of the mechanisms of the complex interactions between pathogenic bacteria, the host immune system, and relevant microbiota, could support the development of new potentially life-saving therapeutic strategies that either enhance the ability of our immune system to eliminate infective bacteria, or to maximize the ability of our microbiota to outcompete foreign pathogens.
